# Entry into primary care-based buprenorphine treatment is associated with identification and treatment of other chronic medical problems

**DOI:** 10.1186/1940-0640-7-22

**Published:** 2012-10-29

**Authors:** Theresa A Rowe, Janet S Jacapraro, Darius A Rastegar

**Affiliations:** 1 Yale University School of Medicine, New Haven, CT, USA; 2 Health Care for the Homeless, University of Maryland School of Medicine, Baltimore, MD, USA; 3 Johns Hopkins University School of Medicine, Baltimore, MD, USA

## Abstract

**Background:**

Buprenorphine is an effective treatment for opioid dependence that can be provided in a primary care setting. Offering this treatment may also facilitate the identification and treatment of other chronic medical conditions.

**Methods:**

We retrospectively reviewed the medical records of 168 patients who presented to a primary care clinic for treatment of opioid dependence and who received a prescription for sublingual buprenorphine within a month of their initial visit.

**Results:**

Of the 168 new patients, 122 (73%) did not report having an established primary care provider at the time of the initial visit. One hundred and twenty-five patients (74%) reported at least one established chronic condition at the initial visit. Of the 215 established diagnoses documented on the initial visit, 146 (68%) were not being actively treated; treatment was initiated for 70 (48%) of these within one year. At least one new chronic medical condition was identified in 47 patients (28%) during the first four months of their care. Treatment was initiated for 39 of the 54 new diagnoses (72%) within the first year.

**Conclusions:**

Offering treatment for opioid dependence with buprenorphine in a primary care practice is associated with the identification and treatment of other chronic medical conditions.

## Background

Opioid and other drug dependence are chronic medical conditions, which can be treated in an outpatient primary care setting as other illnesses are [1]. A number of studies have demonstrated the safety and efficacy of office-based treatment of opioid dependence with sublingual formulations of buprenorphine [2-6]. Potential advantages of office-based treatment over traditional methadone programs include feasibility of implementation in outpatient practices [7]; recruitment of patients who have been reticent to join methadone programs due to expense, need for more frequent attendance, and associated stigma [8]; improved patient retention in treatment [9-11]; and increased patient satisfaction [12].

Individuals with drug dependence have a high burden of medical and psychiatric illness [13-15]. Integration of drug treatment and primary care services may also facilitate the identification and treatment of other chronic medical conditions. Previous studies have shown that providing on-site medical care with drug treatment is associated with improved follow-up with care [16], improved drug outcomes [17,18], and a reduction in emergency department use and hospitalization [19,20]. However, there have been few studies of the medical care of patients engaged in office-based buprenorphine treatment. We undertook this study to evaluate the identification and treatment of other chronic medical conditions among patients who initiated office-based buprenorphine treatment in an academic primary care practice.

## Methods

Comprehensive Care Practice is a primary care practice on the campus of The Johns Hopkins Bayview Medical Center in Baltimore, Maryland, USA (an academic hospital center). The practice is staffed by five attending physicians who are certified to prescribe buprenorphine, three resident physicians who share a panel of patients, and a nurse practitioner. All of the practitioners provide primary care, with a concentration on caring for patients with substance use disorders and HIV infection. It is the policy of the practice that buprenorphine treatment is provided as part of primary care, and buprenorphine is only prescribed to patients who also receive their primary care at this practice.

A cohort of 255 patients who had been given at least one prescription for sublingual buprenorphine from August 2003 until September 2007 had been previously identified, and their drug treatment outcomes have been reported [21]. For this study, we included the subset of patients who had requested treatment for opioid dependence on their initial visit at the practice and had been prescribed the sublingual formulation of buprenorphine or buprenorphine/naloxone within a month of their initial visit.

Data were collected retrospectively from the patient medical records. Both paper charts and electronic medical chart were reviewed, and a data abstraction instrument was used to standardize data collection. Demographic information recorded included age, gender, and type of insurance. The standard initial history and physical form used by this practice includes a page on drug and alcohol use history, prior injection drug use, addiction treatment, and complications of drug and alcohol use. Substance use history collected included substances used and past/current injection drug use. We also recorded known chronic medical conditions that patients reported at the time of the initial visit and were documented in the history and at the physical. In addition, we recorded any information documented regarding treatment of these known medical conditions, including active medication lists and current specialty care. Patients are routinely asked if they have a current or recent primary care provider, and records are requested at the initial visit. We recorded whether patients reported an established relationship with a primary care provider at the time of their initial visit. We reviewed records from the first four months after the initial visit to record any chronic medical conditions that were identified during this period and reviewed records for up to a year to see if any treatment was initiated for these problems. Treatment included counseling (if documented in the patient’s chart), prescribed medication, or referrals for specialty care. All chart reviews were performed by the first author (TAR); when there were questions about classification, these were reviewed with the senior author (DAR), and decisions were made by consensus.

Data analysis was primarily descriptive; we used chi-squared analysis to compare the identification of new medical problems among those who had an established primary care provider at the initial visit with those who did not. This study was approved by the institutional review board of Johns Hopkins University.

## Results

One hundred eighty-nine new patients had come to the practice to receive treatment for opioid dependence and received a prescription for buprenorphine within a month of their initial visit. Of these, 168 (88.9%) patient charts were reviewed; 21 charts were not reviewed due to gaps in identification of subjects or incomplete or inaccessible medical records. Table 1 provides demographic data on the cohort. The mean age was 40 (range, 20 to 64 years). About half (52%) had commercial insurance at their initial visit; 26% had Medicaid, 23% had Medicare, and 3% had no insurance. Fifty-three percent were employed, 24% were unemployed, and 23% were disabled. Over half the patients (54%) reported past injection drug use. Eighty percent reported using heroin, and 36% reported using prescription opioids (16% reported using both); 55% reported cocaine use. Forty-six patients (27%) identified an established primary care provider at the time of their initial visit.

**Table 1:** Demographic information at initial visit

**Characteristic**	**Number** (**total** = **168**)
Mean Age (range)	40 (20–64)
Male	98 (58%)
Female	70 (42%)
Insurance	
Commercial	87 (52%)
Medicaid	43 (26%)
Medicare	33 (20%)
None	5 (3%)
Employment status	
Employed	89 (53%)
Unemployed	40 (24%)
Disabled	39 (23%)
Injection drug use (ever)	91 (54%)
Drugs used	
Heroin	134 (80%)
Prescription opioids	60 (36%)
Cocaine	92 (55%)
Established primary care physician at initial visit	46 (27%)

At least one new chronic medical condition was identified in 47 patients (28%) during the first four months of care. Table 2 provides data on the new diagnoses. A total of 54 new diagnoses were identified; the most common were hypertension (n = 15), hepatitis C (n = 12), hyperlipidemia (n = 10), psychiatric disorders (n = 9), and diabetes mellitus (n = 3). Treatment was initiated for 39 of these new diagnoses (72%) within the first year. Twenty new diagnoses were treated with prescription medications, and two subjects received specialty referrals. Of the 46 subjects who reported having a primary care provider at their initial visit, 15 (32.6%) had a new diagnosis in the first four months compared with 32 (26.2%) of the 122 subjects who did not; this difference was not significant (p = 0.44).

**Table 2:** **New and established diagnoses**, **treatment status at initial visit**, **and initiation of treatment within one year**

**Diagnosis**	**Established Diagnoses (%)**	**New Diagnoses (%)**	**Receiving Treatment at Initial Visit (% of established diagnoses)**	**Treatment Initiated**: **new and established diagnoses (%)**
Psychiatric	78 (46%)	9 (5%)	33 (20%)	44 (77%)
Hepatitis C	62 (37%)	12 (7%)	0 (0%)	2 (3%)
Hypertension	28 (17%)	15 (9%)	10 (36%)	32 (76%)
Hyperlipidemia	9 (5%)	10 (6%)	4 (44%)	14 (74%)
HIV	7 (4%)	0 (0%)	1 (14%)	2 (29%)
Diabetes	4 (2%)	3 (2%)	2 (50%)	5 (71%)
Other	27 (16%)	5 (3%)	19 (11%)	11 (34%)
Total	215 (74%*)	54 (28%*)	69 (32%)	110 (41%)

*Percentage with one or more diagnoses.

One-hundred twenty-five subjects (66%) had at least one chronic medical condition identified at the first visit. Table 2 provides data on the previously identified chronic medical conditions and whether the patient was on treatment at the initial visit. A total of 215 established diagnoses were documented on the initial visit, of which 146 (68%) were not being actively treated at that time. The most common diagnoses were psychiatric disorders (n = 78), hepatitis C (n = 62), hypertension (n = 28), hyperlipidemia (n = 9), and HIV infection (n = 7). Seventy of the previously untreated established diseases (43%) were treated within one year.

Overall, as shown in Table 2, treatment was initiated for 110 (41%) of the established or newly diagnosed conditions. Although only 69 (32%) of the 215 previously identified conditions were being actively treated at the time of initial presentation, by the end of the first year after initiation of office-based buprenorphine treatment, 168 (66%) of the 269 established or newly diagnosed conditions had been addressed with counseling, medical treatment, or referral to specialty care.

## Discussion

Office-based buprenorphine treatment has been shown to be safe and effective for treatment of opioid dependence and allows for integration of treatment into the primary care setting [2-4]. Our own experience with treating patients has likewise been positive in terms of drug treatment outcomes [21]. This study adds to our previous findings by illustrating how treatment of opioid dependence in a primary care setting offers the additional benefit of facilitating the identification and treatment of other chronic medical conditions. Two-thirds of the patients presenting to this practice for treatment of opioid dependence had at least one other known chronic medical condition documented at presentation, and at least one new chronic medical condition was diagnosed in more than a quarter of the subjects. Most did not report an established source for primary care. If these patients had sought treatment at a drug treatment program that did not also provide primary care, these medical conditions might not have been identified. In addition to identification of chronic medical conditions, we found that most patients received some form of treatment for their previously identified or newly diagnosed conditions, suggesting that treatment of drug dependence in the primary care setting engages patients in longitudinal care and facilitates the ongoing management of other chronic medical problems. Providing buprenorphine treatment in a primary care setting is congruent with the move toward the patient-centered medical home model and has the potential for engaging and improving the health of a vulnerable and historically underserved population.

There are few studies looking at the treatment of other chronic medical conditions among patients receiving office-based buprenorphine. One study of a cohort of 228 patients beginning buprenorphine treatment reported that most had medical or psychiatric comorbidities that were being co-managed along with their addiction treatment [22]. Most of the attention to date has been paid to integrating office-based buprenorphine with the treatment of HIV infection with antiretroviral therapy (ART) [23,24]. A recent study by Altice and colleagues [25] found that initiating buprenorphine in an HIV clinical care setting was associated with initiation of ART and improved CD4 lymphocyte counts. Previous studies with methadone maintenance subjects have found that providing on-site medical care resulted in improved follow-up with medical care [16] and reduced use of acute health care services [26]. Further research is needed to look at the effect on clinical outcomes and to determine optimal models of care.

There are a number of limitations to our study. We relied on chart review and documentation by the clinicians, who themselves relied on patient reports. It is possible that some of these problems had been previously identified and treated, but these were not reported by the patient or documented by the clinician. On the other hand, previous studies have found medical record review to be fairly accurate compared with other methods of gathering information [27]. In addition, if a current or recent relationship with a primary care provider was not documented on the initial history and physical form, we counted them as not having an established health care provider. Thus, we may have underestimated the number of patients with previously established primary care.

The small sample size and relatively small number of diagnoses are further limitations. Another limitation is that there was no comparison group; it is possible that these problems may have been identified and these subjects may have received treatment even if they had not initiated treatment or had sought treatment in another setting. Finally, we did not investigate clinical outcomes of these patients. There are almost certainly ways in which their general medical care could be improved further; for example, it is notable that few of the subjects received treatment for hepatitis C infection.

In summary, we found that initiation of office-based buprenorphine treatment in a primary care setting is associated with identification and treatment of other chronic medical problems, suggesting a benefit beyond drug treatment outcomes.

## Competing interests

The authors declare that they have no competing interests.

## Authors’ contributions

TR performed data collection, participated in design of the study, and helped draft the manuscript. DR participated in design of the study, performed the statistical analysis, and helped with manuscript preparation. JJ participated in primary data collection and review of the manuscript. All authors read and approved the final manuscript.
